# Dual orexin receptor antagonists show distinct effects on locomotor performance, ethanol interaction and sleep architecture relative to gamma-aminobutyric acid-A receptor modulators

**DOI:** 10.3389/fnins.2013.00254

**Published:** 2013-12-24

**Authors:** Andres D. Ramirez, Anthony L. Gotter, Steven V. Fox, Pamela L. Tannenbaum, Lihang Yao, Spencer J. Tye, Terrence McDonald, Joseph Brunner, Susan L. Garson, Duane R. Reiss, Scott D. Kuduk, Paul J. Coleman, Jason M. Uslaner, Robert Hodgson, Susan E. Browne, John J. Renger, Christopher J. Winrow

**Affiliations:** ^1^Merck Research Laboratories, Department of Neuroscience, Merck & Co., Inc.West Point, PA, USA; ^2^Merck Research Laboratories, Department of In Vivo Pharmacology, Merck & Co., Inc.West Point, PA, USA; ^3^Merck Research Laboratories, Department of Medicinal Chemistry, Merck & Co., Inc.West Point, PA, USA

**Keywords:** orexin, dual orexin receptor antagonist, locomotor performance, sleep, insomnia, GABA, ethanol, rats

## Abstract

Dual orexin receptor antagonists (DORAs) are a potential treatment for insomnia that function by blocking both the orexin 1 and orexin 2 receptors. The objective of the current study was to further confirm the impact of therapeutic mechanisms targeting insomnia on locomotor coordination and ethanol interaction using DORAs and gamma-aminobutyric acid (GABA)-A receptor modulators of distinct chemical structure and pharmacological properties in the context of sleep-promoting potential. The current study compared rat motor co-ordination after administration of DORAs, DORA-12 and almorexant, and GABA-A receptor modulators, zolpidem, eszopiclone, and diazepam, alone or each in combination with ethanol. Motor performance was assessed by measuring time spent walking on a rotarod apparatus. Zolpidem, eszopiclone and diazepam [0.3–30 mg/kg administered orally (PO)] impaired rotarod performance in a dose-dependent manner. Furthermore, all three GABA-A receptor modulators potentiated ethanol- (0.25–1.5 g/kg) induced impairment on the rotarod. By contrast, neither DORA-12 (10–100 mg/kg, PO) nor almorexant (30–300 mg/kg, PO) impaired motor performance alone or in combination with ethanol. In addition, distinct differences in sleep architecture were observed between ethanol, GABA-A receptor modulators (zolpidem, eszopiclone, and diazepam) and DORA-12 in electroencephalogram studies in rats. These findings provide further evidence that orexin receptor antagonists have an improved motor side-effect profile compared with currently available sleep-promoting agents based on preclinical data and strengthen the rationale for further evaluation of these agents in clinical development.

## Introduction

Orexin neurons are a cluster of cells located in the perifornical lateral hypothalamic area. The neuropeptides orexin A and B bind to and activate the orexin 1 and 2 receptors (OX_1_R and OX_2_R, respectively) with differential selectivity; orexin A binds both receptors with similar affinity while orexin B exhibits approximately 10-fold selectivity for OX_2_R (Sakurai et al., 1998). Recent advances have been made in the development of pharmacological agents that antagonize orexin receptors and interest in their potential clinical application has increased (Scammell and Winrow, 2011). The most salient role for orexin signaling is its regulation of arousal and vigilance stage control, but it has also been implicated in several other processes including feeding, reward, stress, and mood (Gotter et al., 2012). The neuronal networks underlying these processes are becoming better understood as orexin receptor antagonists with greater selectivity have been developed (Ohno and Sakurai, 2008).

Orexin neurons are active during the waking phase in animals, but are largely quiescent during the sleep phase (Yoshida et al., 2001; Kiyashchenko et al., 2002). Genetic loss of orexin signaling in canines and rodents and orexin deficiency in humans causes symptoms of narcolepsy (Chemelli et al., 1999; Lin et al., 1999; Nishino et al., 2000), indicating the importance of orexin neurons in regulating sleep-arousal mechanisms. For example, damaging orexin neurons using the neurotoxin hypocretin-2-saporin alters sleep behavior in rats (Gerashchenko et al., 2003) and acute optogenetic silencing of these cells *in vivo* induces slow-wave sleep in mice (Tsunematsu et al., 2011). Orexin neurons of the lateral hypothalamus project to nuclei involved in regulating arousal and vigilance state. OX_1_R and OX_2_R have overlapping expression in these regions, with OX_2_R having selective expression in tuberomammillary nuclei of the hypothalamus, primarily responsible for driving histamine-mediated arousal (Lin et al., 1988; Trivedi et al., 1998; Marcus et al., 2001). Both receptors are expressed in brainstem nuclei involved in sleep regulation, the exception being the selective expression of OX_1_R in the locus coeruleus, most notably involved in the regulation of REM sleep (Trivedi et al., 1998; Marcus et al., 2001). Regulation of orexin neuron activity is provided both by excitatory projections from the dorsomedial nucleus of the hypothalamus and by inhibitory gamma-aminobutyric acid (GABA) inputs arising from the ventrolateral preoptic area (Thompson et al., 1996; Yoshida et al., 2006). Increased inhibitory GABA activity on orexin neurons and other sleep-related nuclei suppresses arousal and promotes sleep (Saper et al., 2005).

Currently available sleep agents (including benzodiazepines and related compounds) are typically GABA-A receptor modulators, which are believed to mediate sedative effects through their high affinity for the GABA-A α1 receptor subunit (Graham et al., 1996; Rudolph et al., 1999; McKernan et al., 2000). Hypnotics do not bind to the GABA-A receptor active site but instead potentiate the affinity of GABA for the receptor via allosteric modulation and result in increased inhibitor activity of the receptor (Bianchi, 2010). GABA-A receptors are located throughout the central nervous system (CNS), and have a role not only in sleep induction, but in numerous other brain functions as well (Sieghart and Sperk, 2002). This broad functionality can lead to a variety of undesirable side effects such as unsteady gait, next-day sedation, cognition deficits, lost balance and confusion (Barker et al., 2004; Hindmarch et al., 2006; Otmani et al., 2008; Vermeeren and Coenen, 2011). In an attempt to boost their sleep-promoting effects, some chronic users of GABA-A receptor modulators combine these agents with alcohol (Johnson et al., 1998).

Ethanol acts, at least partially, by enhancing GABA transmission at GABA-A receptors (Hanchar et al., 2005; Lovinger and Roberto, 2013) and chronic consumption of alcohol can result in cross-tolerance to benzodiazepines (Devaud et al., 1996; Cagetti et al., 2003). The actions of alcohol and benzodiazepines therefore converge mechanistically at GABA-A receptors, synergistically increasing their activity and potentially amplifying next-day hangover effects. It is well-documented that co-administration of alcohol and benzodiazepines/benzodiazepine receptor modulators can precipitate toxicities and accidents (Tanaka, 2002; Kurzthaler et al., 2005), emphasizing the unmet need for novel sleep-promoting agents with improved adverse-event profiles.

Recently, a number of orexin receptor antagonists including SB-649868 (Bettica et al., 2012a), almorexant (Brisbare-Roch et al., 2007), suvorexant (Winrow et al., 2011) and MK-6096 (Winrow et al., 2012) have demonstrated sleep-promoting properties in both animal and in clinical studies (Herring et al., 2012a; Hoever et al., 2012; Bettica et al., 2012b). As these antagonists target orexin receptors directly, we were interested in comparing structurally distinct orexin receptor antagonists with a variety of GABA-A receptor modulators, to evaluate their effects on sleep architecture and potential for ethanol interactions. In foundational studies, Steiner et al. (Steiner et al., 2011) compared the effects of almorexant and zolpidem both alone and when co-administered with ethanol in the context of rat locomotor performance and myorelaxation in rotarod and grip strength assays. Zolpidem affected both locomotor performance and grip strength in a dose-dependent manner and was exacerbated by ethanol, whereas neither of these changes were observed with almorexant at dosages up to 300 mg/kg (PO). The earlier study examined one representative compound from each class, specifically in rotarod and grip assays. In order to make a thorough assessment of the mechanistic differences between the two drug classes, the current study evaluated the locomotor performance and ethanol interaction not only of almorexant, but also a chemically distinct dual orexin receptor antagonists (DORA) of diverse structure, DORA-12, as well as benzodiazepine and non-benzodiazepine GABA-A modulators diazepam, eszopiclone and zolpidem. Further, locomotor performance was evaluated directly in the context of sleep-promoting efficacy. These results demonstrate a distinct difference between these sleep-promoting mechanisms, orexin antagonism and GABA-A receptor activation, in terms of locomotor performance in the presence or absence of ethanol and in the context of effects on sleep architecture.

## Materials and methods

### Animals

All animal studies were performed in accordance with the National Research Council Institute of Laboratory Animal Resources Guide for the Care and Use of Laboratory Animals and were approved by the Merck Institutional Animal Care and Use Committee. Male Sprague-Dawley rats (180–225 g) were maintained under normal laboratory conditions (controlled temperature and food plus water *ad libitum*) under a regular 12-h light/dark cycle (18:00 lights off and 06:00 lights on). Male animals were used to avoid variability due to time-dependent changes in estrus and rat sex-dependent differences in metabolic enzyme activity. All behavioral tests were performed during the light phase of the cycle, which in nocturnal rats is the normal resting phase. Animals were fasted overnight, housed two per cage prior to testing and acclimated to the testing room for 30 min before testing. All studies used a between-subjects design.

### Compounds and formulations

All pharmacological agents were synthesized by Merck chemists or purchased from Sigma Company (Cream Ridge, NJ, USA) and diluted in 20% Vitamin E-TPGS (d-alpha-tocopheryl polyethylene glycol 1000 succinate) vehicle to a dose volume of 2 mL/kg, to be administered orally using standard stainless steel gavage needles affixed to a 5 cc syringe (PO). Ethanol was diluted in distilled water to a dose volume of 2 mL/kg to be administered intra-peritoneally (IP). After the acclimation period, rats were administered compound or vehicle PO and tested after 30 min or were administered compound PO followed immediately by ethanol IP and tested after 30 min.

### Rotarod test

The rotarod apparatus (IITC Life Sciences, Woodland Hills, CA, USA) consisted of a rod 60 mm in diameter suspended 25.4 cm from the floor of the apparatus. The speed of the rod could be varied as desired. Rats were first trained over 2 days (2–3 trials) to walk on the rod rotating at a constant speed (12 rpm). Rats were then selected for inclusion in assays if they were able to remain on the rod for 120 s as it was accelerated from 4 to 20 rpm (over 150 s). Animals unable to perform the training task were excluded from further testing. Roughly 10% of the animals failed to meet this criterion.

During compound testing, rats were administered the test compound and then returned to their home cage for the duration of the pretreatment period. The rats were then placed on the rotarod at a speed that accelerated from 4 to 40 rpm over 300 s. Latency to fall (time to the rat falling from the apparatus, up to 300 s) for each animal was digitally recorded by trip plates in the platform which were triggered as soon as the rat fell from the rod. One testing trial was performed for each animal.

### Plasma concentrations and ethanol blood levels

At the end of the rotarod testing, which lasted approximately 35–40 min, rats were immediately euthanized by carbon dioxide overdose and blood samples were drawn via cardiac puncture for compound level and/or blood alcohol content (BAC) testing. Blood was centrifuged at 1300 RCF for 10 min at 4°C to obtain serum for determination of compound plasma concentration and BAC. Ethanol analysis was performed using the Siemens Ethanol_2 method on the Siemens Advia 1800 clinical chemistry analyzer. The method utilizes alcohol dehydrogenase to catalyze the oxidation of ethanol to acetaldehyde as well as reducing nicotinamide adenine dinucleotide (NAD) to NADH. This causes an increase in absorbance at 340 nm which is proportional to the BAC (Clinical Pathology Diagnostic Group at Merck West Point).

Data were analyzed using a one-way analysis of variance followed by a *post-hoc* Newmans-Keuls multiple comparison test; *p* < 0.05 was considered significant. Data in the results tables were normalized to controls.

### Rat ambulatory polysomnography

Male Sprague-Dawley rats (Charles Rivers Laboratory, Raleigh, NC; *n* = 8–16/study, weight: 450–600 g) were singly housed in polycarbonate cages (48.3 × 25.4 × 20.3cm; LabProducts, Seaford, DE) with free access to food and water in a 12:12 light:dark cycle. Prior to testing, adult rats of sufficient weight were implanted with radio telemetric devices (TL10M3-F50-EEE, Data Sciences International, Arden Hills, MN) and mean time in vigilance states [active wake, light sleep, delta sleep, rapid eye movement (REM) sleep] was determined as detailed previously (Renger et al., 2004; Winrow et al., 2011, 2012). For these experiments, vehicle and drug treatments were administered on three consecutive days during the middle of the active or dark phase (Zeitgeber Time 18:00–18:30) in a balanced cross-over design such that each animal received both vehicle and drug in alternate arms of the experiment. Mean within-subject change from vehicle was determined for time spent in each vigilance state for the 2 h following treatment. Population *t*-tests were used to determine significance from vehicle under each condition.

## Results

### Individual acute effects on motor performance of GABA-A receptor modulators, ethanol and orexin receptor antagonists

**Figure 1:**
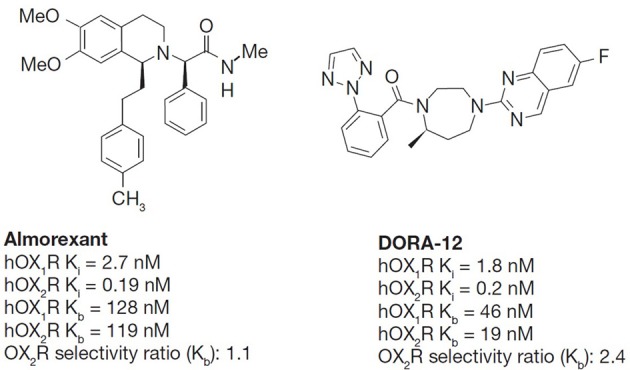
**The chemical structures of almorexant and DORA-12**. Both almorexant and DORA-12 have similar antagonistic activity on both orexin receptors with slight OX_2_R selectivity in affinity. *K_i_*, competitive inhibition binding constant determined in ligand displacement assays of Chinese hamster ovary (CHO) cell membranes expressing recombinant human OX_1_R and OX_2_R, K^b^, functional inhibition of OX-A[Ala6,12]-induced calcium mobilization in CHO cells expressing human OX_1_R and OX_2_R in fluorimetric imaging plate reader (FLIPR) assays. The *in vitro* potency of DORAs, including almorexant, for OX_1_R and OX_2_R is consistent across species and less than 2-fold differences are observed between human and rat receptors (Winrow et al., 2012).

The chemical structures of the orexin receptor antagonists included in this study are shown in Figure 1.

**Figure 2:**
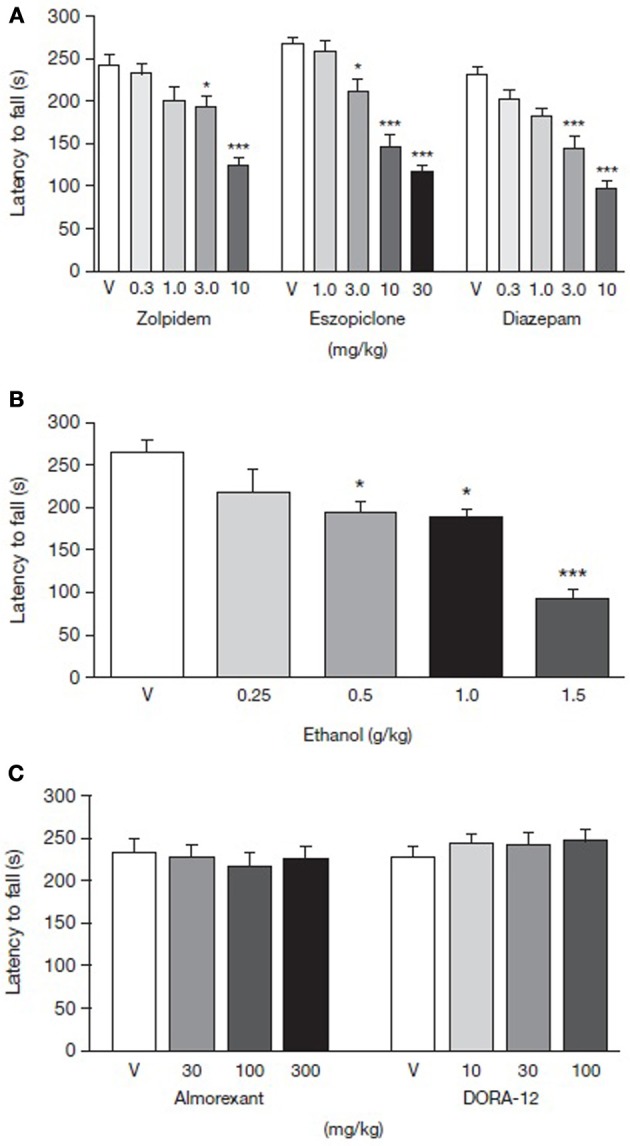
**Dose-response curve for latency to fall from rotarod for rats administered (A) GABA-A receptor modulators, (B) ethanol, and (C) orexin receptor antagonists**. Comparison of GABA-A receptor modulators, ethanol and orexin receptor antagonists in terms of latency to fall using rotarod as a measure of motor performance. Paraoral acute administration of zolpidem, eszopiclone, diazepam, and IP administration of ethanol dose-dependently impairs rotarod performance. Paraoral doses of orexin receptor antagonists, almorexant and DORA-12, do not impair locomotor performance after acute administration. Data are shown as mean ± standard error of the mean. ^*^p < 0.05, ^***^p < 0.001 for the level of significance vs. the vehicle-treated group. ANOVA *post-hoc* Newman-Keuls multiple comparison tests (*n* = 7–12). ANOVA, analysis of variance; PO, per oral; V, vehicle.

Time spent walking on the rotarod by rats treated with either GABA-A receptor modulators, orexin receptor antagonists, ethanol or vehicles was measured to assess the compounds' individual effects on motor performance. Rotarod performance 30 min after drug administration was impaired in a dose-dependent manner by all the GABA-A receptor modulators tested (Figure 2A). Zolpidem [*F*_(4, 38)_ = 14.26, *p* < 0.01], eszopiclone [*F*_(4, 45)_ = 38.19, *p* < 0.01] and diazepam [*F*_(4, 44)_ = 18.1, *p* < 0.01] all had a significant effect on rotarod performance. The highest doses of zolpidem, eszopiclone and diazepam (10, 30, and 10 mg/kg, respectively) reduced motor ability by 48.2, 56.1, and 57.8%, respectively, compared with vehicle treatment (*p* < 0.001; Table 1). The lowest dose to result in significant impairment compared with vehicle-treated animals was 3.0 mg/kg for all of the GABA-A receptor modulators [19.7, 15.1, and 30.2% impairment for zolpidem, eszopiclone, and diazepam, respectively; *p* < 0.01 for all (Table 1)].

**Table 1:** **Dose-response effects of ethanol, GABA-A receptor modulators and orexin receptor antagonists on compound plasma or blood alcohol concentrations and latency to fall from the rotarod**.

**Treatment**	**Dose^a^**	**[Compound]_plasma_/BAC(μM) / (mg/dL)^b^**	**Rotarod performance (% change)**	**Latency to fall (s)^b^**
**ETHANOL TESTS**				
Vehicle	V	−	0	264.8 ± 15.5
Ethanol	0.25	<10	−17.2	219.1 ± 27.6
	0.50	35.4 ± 4.8	−26.2^*^	195.4 ± 12.9^*^
	1.0	107.0 ± 8.8	−28.2^*^	189.7 ± 9.9^*^
	1.5	204.1 ± 11.3	−64.6^***^	93.7 ± 10.6^***^
**ZOLPIDEM TESTS**				
Vehicle	V	−	0	242.5 ± 12.3
Zolpidem	0.3	0.036 ± 0.004	−3.7	233.6 ± 10.4
	1.0	0.115 ± 0.020	−16.9	201.4 ± 15.9
	3.0	0.333 ± 0.040	−19.7^*^	194.6 ± 11.0^*^
	10	0.878 ± 0.121	−48.2^***^	125.6 ± 8.4^***^
**ESZOPICLONE TESTS**				
Vehicle	V	−	0	267.9 ± 6.9
Eszopiclone	1.0	0.090 ± 0.01	−3.4	258.9 ± 12.1
	3.0	0.400 ± 0.03	−15.1^*^	212.6 ± 8.2^*^
	10	1.100 ± 0.12	−45.1^***^	147.1 ± 13.0^***^
	30	3.15 ± 0.53	−56.1^***^	117.5 ± 7.0^***^
**DIAZEPAM TESTS**				
Vehicle	V	−	0	231.8 ± 26.5
Diazepam	0.3	0.04 ± 0.01	−8.4	203.6 ± 10.4
	1.0	0.05 ± 0.02	−15.7	182.3 ± 9.6
	3.0	0.13 ± 0.08	−30.2^***^	145.1 ± 13.9^***^
	10	0.31 ± 0.15	−57.8^***^	97.8 ± 7.9^***^
**ALMOREXANT TESTS**				
Vehicle	V	−	0	235.4 ± 16.3
almorexant	30	0.62 ± 0.12	−2.6	229.3 ± 14.0
	100	4.81 ± 0.37	−7.1	218.7 ± 14.0
	300	11.81 ± 1.38	−3.2	227.9 ± 13.0
**DORA-12 TESTS**				
Vehicle	V	−	0	229.0 ± 13.3
DORA-12	10	0.43 ± 0.04	+7.4	246.0 ± 9.8
	30	0.97 ± 0.09	+6.4	243.6 ± 10.2
	100	4.06 ± 0.40	+8.3	247.9 ± 13.8

ANOVA, analysis of variance; SEM, standard error of the mean; V, vehicle

Plasma/alcohol levels refer to time points between 35 and 40 min after dosing of compounds.

a Doses of zolpidem, eszopiclone and diazepam are given in mg/kg; doses of ethanol are given as g/kg.

b Mean ± SEM shown for compound concentrations in plasma, blood alcohol concentrations (BAC) and Latency to fall.

* p < 0.05,

*** p < 0.001 significance versus vehicle treatment group. ANOVA post-hoc Newman-Keuls multiple comparison test.

Ethanol administration dose-dependently impaired motor performance in the rats [*F*_(4, 30)_ = 11.83, *p* < 0.01] with doses of 0.5, 1.0 and 1.5 g/kg significantly decreasing latency to fall relative to vehicle (Figure 2B). The highest ethanol dose (1.5 g/kg) produced a 64.6% decrease in motor ability in the rotarod compared with vehicle-treated animals (*p* < 0.001, Table 1).

Almorexant doses ranging from 30 to 300 mg/kg PO did not affect the rats' ability to walk on the rotarod [*F*_(3, 36)_ = 0.23, *p* > 0.05] (Figure 2C and Table 1). Similarly, DORA-12 did not affect rotarod performance over doses ranging from 10 to 100 mg/kg, PO [*F*_(3, 38)_ = 0.54, *p* > 0.05] (Figure 2C and Table 1).

BAC and compound plasma concentrations were monitored to ensure that levels increased concomitantly with increasing doses of ethanol or sleep-promoting compounds, respectively. Plasma levels of the GABA-A receptor modulators increased with higher doses (Table 1) and were accompanied by a greater reduction in latency to fall. Similarly, BAC increased with increased doses of ethanol (Table 1). In contrast, latency to fall was not reduced by increasing doses of almorexant and DORA-12, despite concomitant increases in the plasma concentrations of these orexin receptor antagonists (Table 1 and Figure 2).

### Differential sleep-promoting effects of ethanol, GABA-A receptor modulators and DORA-12

**Figure 3:**
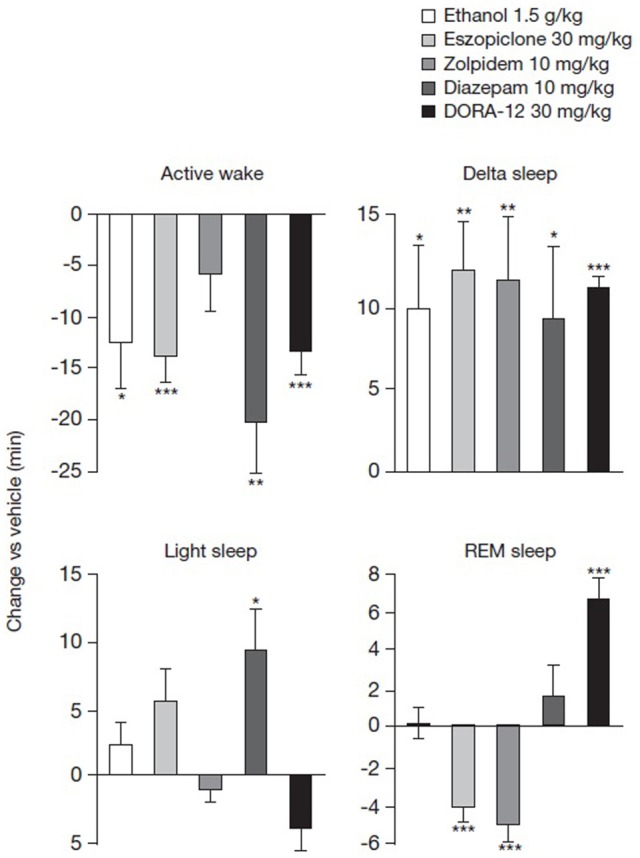
**Differential effects of ethanol, GABA-A receptor modulators and DORA-12 on sleep stages**. Mean time in active wake, delta, light and REM sleep was monitored for 2 h after PO administration of ethanol (1.5 g/kg), eszopiclone (30 mg/kg), zolpidem (10 mg/kg), diazepam (10 mg/kg), or DORA-12 (30 mg/kg). Three consecutive days of each treatment was administered in a balanced cross-over design such that each subject received drug and vehicle. Mean within-subject change relative to vehicle is shown. Data were analyzed using within-subject paired *t*-tests relative to vehicle (*N* = 8–16; ^*^p < 0.05, ^**^p < 0.01, ^***^p < 0.001). PO, paraoral; REM, rapid eye movement.

To evaluate the effects of these compounds on locomotor activity in the context of sleep promotion, the benzodiazepine (diazepam) and non-benzodiazepine (eszopiclone and zolpidem) GABA-A receptor modulators, the orexin receptor antagonist DORA-12 and ethanol, were assessed for their ability to promote sleep in rats at the highest doses tested in rotarod experiments. Almorexant has been well-documented to promote sleep at dosages ranging from 30 to 300 mg/kg, the approximate maximum effect level (Brisbare-Roch et al., 2007), and has been previously shown in our lab to attenuate active wake at 100 mg/kg for as long as 7 h (Gotter et al., 2013). As seen in Figure 3, all treatments with the exception of zolpidem 30 mg/kg significantly attenuated active wake during the 2 h following treatment (−6.0 min ± 3.6 min, *p* = 0.118, paired *t*-test for individual animals relative to vehicle). DORA-12 reduced the time spent in active wake to a similar extent as eszopiclone (30 mg/kg) and ethanol (1.5 g/kg) (reductions of 13.5 ± 2.3, 14.0 ± 2.7, 12.7 ± 4.4 min, respectively), while diazepam 10 mg/kg had the greatest effect (20.4 ± 5.0 min). Relative to vehicle, all treatments significantly increased delta sleep time during the 2-h analysis period to similar magnitudes, with increases ranging from 9.6 ± 4.6 min in the case of diazepam to 12.8 ± 2.9 min for eszopiclone. DORA-12 increased delta sleep by 10.9 ± 1.4 min. Light sleep, which is a minor component of rat sleep, was only significantly affected by diazepam in these studies. The most striking differences were on REM sleep changes. Ethanol and diazepam had no effect on REM, but eszopiclone and zolpidem both attenuated REM sleep relative to vehicle (−4.2 ± 0.8 and −5.1 ± 0.9 min, respectively), despite the fact that zolpidem was not effective in significantly reducing active wake in this experiment. DORA-12, on the other hand, significantly increased REM sleep time relative to vehicle (by 6.6 ± 1.1 min). We have recently shown that almorexant, dosed at 100 mg/kg during the active phase, has similar effects in terms of the magnitude of active wake reduction as a 30 mg/kg dose of DORA-12 (Gotter et al., 2013), consistent with previous observations (Brisbare-Roch et al., 2007). These results demonstrate a clear difference between the sleep promoted by the standard of care for insomnia and the effects of DORA-12.

### Acute effect on motor performance of GABA-A receptor modulators in combination with ethanol

Sub-threshold doses (below the doses observed to impair motor performance) of the GABA-A receptor modulators zolpidem, eszopiclone, and diazepam were identified from their respective dose–response curves (Figure 2A). A minimum effective dose of ethanol (1 g/kg) was identified that produced BAC ranging from 106.9 ± 7.7 mg/dL−139.1 ± 2.42 mg/dL (Figure 2B). Sub-threshold doses of the GABA-A receptor modulators were then co-administered with the minimum effective dose of ethanol to assess whether ethanol may have additive motor impairments associated with GABA-A receptor modulators.

**Figure 4:**
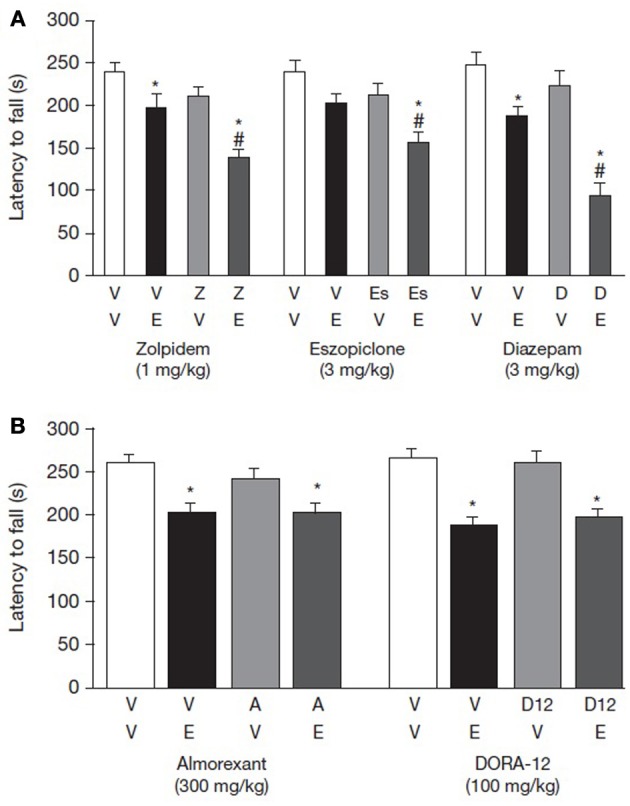
**Interaction of ethanol with (A) GABA-A receptor modulators or (B) orexin receptor antagonists on rotarod performance**. Effect of GABA-A receptor modulators and orexin receptor antagonists on rotarod performance. Effects of zolpidem, eszopiclone, and diazepam were tested using a minimum effective dose (MED) of ethanol and a MED of test compound based on the results of the dose-response rotarod studies. Effects of almorexant and DORA-12 were tested using doses well-above those required to initiate sleep in combination with the MED of ethanol. Data are shown as mean ± standard error of the mean. ^*^p < 0.05 for the level of significance vs. vehicle-vehicle group and ^#^*p* < 0.05 denotes significant difference from the vehicle-ethanol group. Two-Way ANOVA was followed by *post-hoc* tests (Newman-Keuls multiple comparison tests, *n* = 7–12). ANOVA, analysis of variance; D, diazepam; E, ethanol; Es, eszopiclone; V, vehicle; Z, zolpidem.

Diazepam 3.0 mg/kg co-administered with ethanol reduced rats' latencies to fall from the rotarod by 63.4% compared with vehicle (Figure 4A and Table 2). By way of comparison, rats administered ethanol or diazepam alone demonstrated 30.8 and 15.5% decreases, respectively, in latency to fall (Table 2). In these animals, there was a significant main effect of diazepam and ethanol [*F*_(1, 32)_ = 10.8 and 49.7, respectively, *p* < 0.001] as well as a significant diazepam by ethanol interaction [*F*_(1, 32)_ = 4.8, *p* = 0.036]. *Post-hoc* analysis showed that animals administered ethanol alone differed significantly from animals given only vehicle (*p* < 0.05). *Post-hoc* tests also revealed that animals given diazepam with ethanol differed from animals given diazepam or ethanol alone (*p* < 0.05).

**Table 2:** **Interaction of GABA-A receptor modulators and orexin receptor antagonists combined with a minimum effective dose of ethanol on drug plasma concentration, blood alcohol concentrations and latency to fall from the rotarod**.

**Treatment**	**Compound dose (mg/kg)**	**Ethanol dose (g/kg)**	**[Compound]_plasma_ (μM)^a^**	**BAC (mg/dL)^a^**	**% Change in rotarod performance**	**Latency to fall (s)^a^**
**ZOLPIDEM TESTS**						
Vehicle	V	V	–	–	0	242.7 ± 10.0
Zolpidem	1.0	V	0.102 ± 0.02	–	−12.3	212.9 ± 10.9
	V	1.0	–	121.5 ± 14.2	−18.2^*^	198.6 ± 16.2^*^
	1.0	1.0	0.086 ± 0.04	124.4 ± 11.7	−42.3^*^	140.1 ± 9.1^*^^#^
**ESZOPICLONE TESTS**						
Vehicle	V	V	–	–	0	242.9 ± 12.4
Eszopiclone	3.0	V	0.42 ± 0.04	–	−12.1	213.4 ± 15.6
	V	1.0	–	106.9 ± 7.7	−16.3	203.4 ± 11.4
	3.0	1.0	0.20 ± 0.07	114.8 ± 5.4	−34.5^*^	159.0 ± 12.1^*^^#^
**DIAZEPAM TESTS**						
Vehicle	V	V	–	–	0	250.4 ± 13.8
Diazepam	3.0	V	0.103 ± 0.03	–	−15.5	226.5 ± 15.8
	V	1.0	–	139.1 ± 2.42	−30.8^*^	188.7 ± 12.5^*^
	3.0	1.0	0.145 ± 0.04	119.8 ± 12.6	−63.4^*^^#^	94.6 ± 15.7^*^^#^
**ALMOREXANT TESTS**						
Vehicle	V	V	–	–	0	263.4 ± 9.12
Almorexant	300	V	9.67 ± 0.95	–	−7.2	244.3 ± 11.4
	V	1.0	–	109.2 ± 6.7	−22.3	204.7 ± 10.6^*^
	300	1.0	7.77 ± 0.75	105.6 ± 7.12	−2.5	204.2 ± 11.1^*^
**DORA-12 TESTS**						
Vehicle	V	V	–	–	0	267.2 ±12.9
DORA-12	100	V	3.03 ± 0.37	–	−1.5	263.1 ± 13.5
	V	1.0	–	130.6 ± 2.8	−29.1	189.4 ± 10.5^*^
	100	1.0	1.25 ± 0.36	123.5 ± 5.0	−25.1	200.1 ± 9.1^*^

ANOVA, analysis of variance; SEM, standard error of the mean; V, vehicle.

a Mean ± SEM.

* p < 0.05, significance versus vehicle-vehicle;

# p < 0.05, significance versus vehicle-ethanol. Two-Way ANOVA was followed by post-hoc tests (Newman-Keuls multiple comparison test).

A similar pattern of effect was observed with eszopiclone. Both eszopiclone and ethanol produced a main effect of treatment [*F*_(1, 34)_ = 7.78 and 12.58, *p* = 0.001 and 0.009, respectively]; however, there was no interaction when the two compounds were given together, suggesting an additive effect. The percent decrease in rotarod latency when eszopiclone was given without and with alcohol was 12.1% and 34.5%, respectively. *Post-hoc* analysis showed that animals administered ethanol (*p* > 0.05) did not differ significantly from vehicle-vehicle group (Figure 4A and Table 2).

Zolpidem also negatively impacted rotarod performance, as indicated by a main effect of zolpidem treatment [*F*_(1, 29)_ = 14.53, *p* < 0.001]. Ethanol likewise produced an effect in this experiment, as indicated by a main effect [*F*_(1, 29)_ = 25.49, *p* < 0.001]. The interaction between ethanol and zolpidem was not significant, indicating that the effects of these two compounds on rotarod performance was additive [*F*_(1, 29)_ = 1.53, *p* = 0.23]. The percent decrease in rotarod latency when zolpidem was given without and with ethanol was 12.3 and 42.3%, respectively (Figure 4A and Table 2).

### Acute effect on motor performance of orexin receptor antagonists administered in combination with ethanol

By contrast with the GABA-A receptor modulators, neither almorexant nor DORA-12 potentiated motor performance impairment when co-administered with ethanol (Figure 4B and Table 2). In both the almorexant and DORA-12 experiments, ethanol produced a main effect of treatment [*F*_(1, 35 and 36)_ = 7.84 and 36.5, *p* = 0.008 and <0.001, respectively]. However, neither almorexant nor DORA-12 produced a main effect of treatment, and there was no interaction between either of the two DORAs and ethanol. Rotarod performance was impaired to a similar extent when given with and without almorexant (22.5 and 22.3%, respectively). Likewise, rotarod performance was impaired to a similar extent when ethanol was given with and without DORA-12 (25.1 and 29.1%, respectively). BACs were similar when ethanol was administered alone or in combination with either one of the orexin receptor antagonists (Table 2), demonstrating that neither DORA-12 nor almorexant had a measureable impact on ethanol metabolism.

DORA-12 and almorexant plasma concentrations were stable when administered alone but fluctuated when co-administered with ethanol. Co-administration of ethanol also resulted in fluctuations in the drug–plasma concentrations of the GABA-A receptor modulators (Table 2).

## Discussion

The aim of the current study was to evaluate the potential impact of different insomnia therapeutic mechanisms on locomotor coordination in the presence or absence of ethanol relative to doses effective in promoting sleep. The current work goes beyond that of others (Steiner et al., 2011), by evaluating locomotor performance following treatment with multiple, distinct compounds within each class—two orexin receptor antagonists, a benzodiazepine and two non-benzodiazepine GABA-A receptor modulators—in order to determine if these results were due to pathway-dependent effects and not due to individual compound idiosyncrasies. In addition, locomotor impairment was evaluated in the context of sleep-promoting effects and sleep architecture. These results demonstrate that, at equally effective sleep-promoting doses, GABA-A receptor modulators induce substantial locomotor impairment and ethanol interaction relative to that observed in response to DORAs. This analysis also illustrates fundamental differences in sleep architecture induced by orexin receptor antagonists compared with GABA-A receptor modulators.

The use of multiple, pharmacologically distinct compounds from the orexin receptor antagonist and GABA-A receptor modulator classes indicates that the observed differences in locomotor impairment are due to the different sleep-promoting mechanisms. Studies in animals and humans have demonstrated consistent sleep-promoting effects of orexin receptor antagonists with distinct structural and pharmacological properties (Brisbare-Roch et al., 2007; Winrow et al., 2011, 2012; Bettica et al., 2012c; Herring et al., 2012b). Almorexant has been shown previously not to impair locomotor performance or to interact with ethanol (Steiner et al., 2011). We have further demonstrated that DORA-12 did not disrupt locomotor activity in the presence or absence of ethanol. DORA-12 is chemically distinct from almorexant, has greater *in vivo* potency, a distinct pharmacokinetic profile and faster binding kinetics despite similar *in vitro* activity at the receptor (Brisbare-Roch et al., 2007; Gotter et al., 2013). These results indicate that antagonism of orexin signaling promotes sleep, but does not significantly impair motor function. Contrary to the effects seen with orexin receptor antagonists, three different GABA-A receptor modulators of distinct chemical structure—a classic benzodiazepine, diazepam, and the non-benzodiazepines, zolpidem and eszopiclone—all impaired rotarod performance and potentiated the effects of ethanol in the current study. While all three compounds interact with the same allosteric benzodiazepine binding site on GABA-A receptors to increase their activity, the subunit specificity and pharmacokinetic properties of each differ, particularly that of diazepam which has an extended plasma half-life relative to zolpidem and eszopiclone (Graham et al., 1996; Rudolph et al., 1999; McKernan et al., 2000; Bianchi, 2010; Gotter et al., 2013). This impairment is observed across benzodiazepine and non-benzodiazepine compounds and is likely a result of the widespread expression of GABA-A receptors throughout the CNS (Sieghart and Sperk, 2002). Taken together, the results reveal that the distinct effects on locomotor performance mediated by orexin receptor antagonists vs. GABA-A receptor modulators are due to pathway-dependent effects.

The differences between orexin antagonism and GABA-receptor activation on locomotor performance were also highlighted in the context of sleep-promoting efficacy. DORA-12, which promoted sleep at 30 mg/kg to a similar or greater extent relative to high doses of eszopiclone, zolpidem and ethanol, exhibited no locomotor impairment. Even at 100 mg/kg, DORA-12 induced no locomotor impairment or ethanol interaction. Almorexant administered at 300 mg/kg, a dose well-above that required for sleep-promoting effects (Brisbare-Roch et al., 2007; Gotter et al., 2013), also left locomotor performance unaffected. Comparative clinical studies will be needed to confirm the translatability of these differences observed in the rat rotarod assay.

In addition to comparing the magnitude of sleep promotion, as measured by active wake reduction, the current work also demonstrates clear differences in the sleep architecture induced by GABA-A receptor modulators, DORA-12 and ethanol. At the doses tested, both classes of drugs as well as ethanol significantly promoted delta sleep to similar levels in the 2 h following treatment. In the case of zolpidem, this was despite insignificant effects on active wake reduction. This difference relative to prior studies (e.g., Renger et al., 2004) was likely due to the 2-h quantification performed here. The most striking difference between these classes of compounds was their effects upon REM sleep. The highly significant increase in REM induced by DORA-12 was diametrically opposed to the REM-suppressing effects of eszopiclone and zolpidem. These effects were consistent with prior studies separately evaluating other DORAs (Brisbare-Roch et al., 2007; Bettica et al., 2012a; Winrow et al., 2012) and GABA-A modulators (Brunner et al., 1991; Renger et al., 2004). Differential effects on REM sleep were also observed in a recent study comparing several GABA-A modulators with the orexin antagonist DORA-22; the GABA-A receptor modulators eszopiclone and zolpidem were associated with dose-responsive disruptions in EEG spectral profiles during sleep, while DORA-22 produced only marginal changes in EEG measures during compound-induced sleep (Fox et al., 2013). In the present study, diazepam was highly effective in attenuating active wake; however, this GABA-A modulator did not attenuate REM. This may be due to either GABA-A receptor subtype specificity differences from other compounds in this class (Arbilla et al., 1985), or more likely the limited 2-h analysis used here relative to this longer half-life compound, since diazepam has been noted to attenuate REM in prior studies (Renger et al., 2004). Ethanol attenuated active wake and promoted delta sleep to a similar extent as eszopiclone and DORA-12, but neither increased nor decreased REM sleep.

GABA is the principal inhibitory neurotransmitter in the nervous system, and as a consequence, GABA-A receptor modulators impact neurons throughout the CNS and modulate numerous signaling events, many of which are not involved with sleep promotion. Some of these additional CNS activities may be responsible for the unwanted effects associated with GABA-A receptor agonists, including drowsiness at arousal and induction of seemingly non-restorative sleep (Mohler, 2006). Consequently, some patients use alcohol in conjunction with GABA-A receptor modulators in an attempt to assist with falling asleep. Indeed, aged Finnish males who use anxiolytics and sedatives (mainly benzodiazepines) are reportedly more likely to be binge drinkers and heavy alcohol consumers than those who do not take psychotropics (Ilomaki et al., 2008).

Orexin neurons project to many regions in the brain including discrete brain centers that are part of the sleep-arousal system (Peyron et al., 1998; Marcus et al., 2001). Orexin receptor antagonists may therefore become an important new class of therapeutics that treat insomnia using a novel mechanism (Winrow and Renger, 2013). Even though orexin receptor antagonists are effective at promoting sleep at moderate doses, the observation that they did not impair motor performance in rats (as measured on the accelerating rotarod), or exacerbate the motor impairment induced by alcohol, suggests that the potential for negative motor side effects may be lower with orexin receptor antagonists than with GABA-A receptor modulators. The lowest dose of GABA-A receptor modulators to attenuate motor activity significantly on the rotarod was 3 mg/kg for zolpidem, eszopiclone, and diazepam, corresponding with ranges reported by other studies to promote sleep 1–3 mg/kg for zolpidem, 3–10 mg/kg for eszopiclone and 1–3 mg/kg for diazepam (Renger et al., 2004; Fox et al., 2013; Gotter et al., 2013). Neither almorexant nor DORA-12 affected latency to fall from the rotarod at doses ranging from 30 to 300 mg/kg and from 10 to 100 mg/kg, respectively DORA-12.30 mg/kg induced similar or greater sleep-promoting efficacy relative to highly impairing doses of eszopiclone and zolpidem in the current work, and the sleep-promoting effects were consistent with prior experiments (Fox et al., 2013). Given that the animals treated with DORA-12 or almorexant were able to perform without impairment, it appears that these compounds induce sleep without affecting motor co-ordination in rats.

The potential risks associated with combining GABA-A receptor modulators (including benzodiazepines and benzodiazepine receptor agonists) and alcohol are perhaps best underscored by examining the links between their consumption and impaired driving skills. It is well-established that alcohol alone disrupts sleep, and can induce grogginess and decrease alertness (Stein and Friedmann, 2005). Benzodiazepine use is associated with an increased risk of on-the-road traffic accidents (Barbone et al., 1998; Woratanarat et al., 2009; Chang et al., 2013), and individuals combining alcohol with long-acting benzodiazepines are significantly more likely to be unsafe drivers than persons driving under the influence of alcohol alone (Maxwell et al., 2010). Furthermore, studies have demonstrated that drivers involved in motor vehicle accidents often had detectable levels of benzodiazepines alone and in combination with alcohol (Christophersen and Morland, 2008; Ricci et al., 2008; Legrand et al., 2013). Benzodiazepine use also increases the risk of falls in the elderly (Leipzig et al., 1999; Ray et al., 2000; Ensrud et al., 2002; Allain et al., 2005; Titler et al., 2011). When alcohol is combined with hypnotics, the frequency of falls and hip fractures in the elderly increases further (Allain et al., 2005). Interestingly, a recent study of next-day driving performance, balance and cognitive tests in elderly (65–80 years) healthy volunteers found no impairment by the orexin receptor antagonist, suvorexant, whereas the GABA-A receptor modulator, zopiclone, impaired these parameters (Vermeeren et al., 2012).

In conclusion, these results demonstrate distinct differences between the orexin antagonist and GABA-A receptor mechanisms on sleep architecture and locomotor performance, and their interaction with ethanol. When administered at sleep-inducing doses, structurally distinct orexin receptor antagonists had no effect on rat rotarod performance and did not potentiate the effect of ethanol, whereas benzodiazepine and non-benzodiazepine GABA-A receptor modulators impaired locomotor function and exacerbated the impairing effects of ethanol on this task at doses well-below that required to induce sleep. Furthermore, REM sleep differences were observed between these classes of compounds where DORA-12 induced significant increases in REM sleep as opposed to the REM-suppressing effects of eszopiclone and zolpidem. Our results corroborate the findings of Steiner and colleagues (Steiner et al., 2011) who also reported an absence of motor disruption with the orexin receptor antagonist almorexant and a lack of additive effects when almorexant was co-administered with ethanol, and align with data in human subjects demonstrating that almorexant does not potentiate ethanol-induced motor or cognitive deficits (Hoch et al., 2013). Clinical evaluation of the effects of suvorexant and alcohol in combination in humans is currently underway.

## Author contributions

Susan E. Browne, Joseph Brunner, Susan L. Garson, Andres D. Ramirez, Duane R. Reiss, John J. Renger, Jason M. Uslaner, and Christopher J. Winrow conceived, designed or planned the study. Steven V. Fox, Susan L. Garson, Anthony L. Gotter, Robert Hodgson, Terrence McDonald, Andres D. Ramirez, Duane R. Reiss, Pamela L. Tannenbaum and Jason M. Uslaner collected or assembled the data. Susan E. Browne, Steven V. Fox, Susan L. Garson, Robert Hodgson, Andres D. Ramirez, John J. Renger, Jason M. Uslaner, Christopher J. Winrow, and Lihang Yao performed or supervised analyses. Susan E. Browne, Paul J. Coleman, Steven V. Fox, Susan L. Garson, Anthony L. Gotter, Robert Hodgson, Scott D. Kuduk, Terrence McDonald, Andres D. Ramirez, John J. Renger, Pamela L. Tannenbaum, Spencer J. Tye, Jason M. Uslaner, Christopher J. Winrow, and Lihang Yao interpreted the results. Susan E. Browne, Susan L. Garson, Anthony L. Gotter, Robert Hodgson, Andres D. Ramirez, and John J. Renger wrote sections of the initial draft of the manuscript. All authors provided substantive suggestions for revision or critically reviewed iterations of the manuscript and reviewed and approved the final version of the manuscript.

### Conflict of interest statement

All authors are employed by Merck Sharp & Dohme Corp., a subsidiary of Merck & Co., Inc., Whitehouse Station, NJ, USA, and receive salary from, research support from and potentially own stock in Merck & Co., Inc.
